# Risk and Protective Factors in the COVID-19 Pandemic: A Rapid Evidence Map

**DOI:** 10.3389/fpubh.2020.582205

**Published:** 2020-11-24

**Authors:** Rebecca Elmore, Lena Schmidt, Juleen Lam, Brian E. Howard, Arpit Tandon, Christopher Norman, Jason Phillips, Mihir Shah, Shyam Patel, Tyler Albert, Debra J. Taxman, Ruchir R. Shah

**Affiliations:** Sciome LLC, Research Triangle Park, NC, United States

**Keywords:** rapid evidence mapping, COVID-19, risk factors, protective factors, literature screening, disease susceptibility

## Abstract

**Background:** Given the worldwide spread of the 2019 Novel Coronavirus (COVID-19), there is an urgent need to identify risk and protective factors and expose areas of insufficient understanding. Emerging tools, such as the Rapid Evidence Map (rEM), are being developed to systematically characterize large collections of scientific literature. We sought to generate an rEM of risk and protective factors to comprehensively inform areas that impact COVID-19 outcomes for different sub-populations in order to better protect the public.

**Methods:** We developed a protocol that includes a study goal, study questions, a PECO statement, and a process for screening literature by combining semi-automated machine learning with the expertise of our review team. We applied this protocol to reports within the COVID-19 Open Research Dataset (CORD-19) that were published in early 2020. SWIFT-Active Screener was used to prioritize records according to pre-defined inclusion criteria. Relevant studies were categorized by risk and protective status; susceptibility category (Behavioral, Physiological, Demographic, and Environmental); and affected sub-populations. Using tagged studies, we created an rEM for COVID-19 susceptibility that reveals: (1) current lines of evidence; (2) knowledge gaps; and (3) areas that may benefit from systematic review.

**Results:** We imported 4,330 titles and abstracts from CORD-19. After screening 3,521 of these to achieve 99% estimated recall, 217 relevant studies were identified. Most included studies concerned the impact of underlying comorbidities (Physiological); age and gender (Demographic); and social factors (Environmental) on COVID-19 outcomes. Among the relevant studies, older males with comorbidities were commonly reported to have the poorest outcomes. We noted a paucity of COVID-19 studies among children and susceptible sub-groups, including pregnant women, racial minorities, refugees/migrants, and healthcare workers, with few studies examining protective factors.

**Conclusion:** Using rEM analysis, we synthesized the recent body of evidence related to COVID-19 risk and protective factors. The results provide a comprehensive tool for rapidly elucidating COVID-19 susceptibility patterns and identifying resource-rich/resource-poor areas of research that may benefit from future investigation as the pandemic evolves.

## Introduction

Since the emergence of the novel coronavirus (COVID-19) in late 2019, there has been great interest in what can be learned about this virus and what can be done to slow its spread. As COVID-19 has evolved into a worldwide pandemic, the number of COVID-19 publications has grown exponentially. In order to quickly synthesize published research on COVID-19 risk and protective factors in humans, we sought to conduct a rapid Evidence Map (rEM) (1) of literature published between January 1, 2020–April 3, 2020. Our aim was to quickly identify topic areas with the greatest availability of scientific evidence and highlight where gaps in the literature still remain. In recent years, stakeholders from various fields have begun to rely on evidence synthesis tools to summarize scientific data to inform consensus with respect to decision-making in regard to potential health risks (2–5). In particular, the practice of “Evidence Mapping” is increasingly being used to characterize important areas of study relevant to a given topic along with important gaps in the literature (6). Evidence maps result from a systematic search of a broad field and are undertaken to identify gaps in knowledge and guide future research needs. Typically, the results are presented in a user-friendly format, often as a visual figure or graph, or as a searchable database (6). However, constructing detailed evidence maps can be a resource-intensive procedure, thereby limiting their utility for practical implementation, particularly for rapidly evolving topics when the time available to conduct research and generate findings may be especially limited. Given the growing number of COVID-19 studies being published on a daily basis, the ability to efficiently synthesize knowledge that could be used to improve public health is paramount.

The rEM is a relatively new process defined as: a resource-efficient form of knowledge synthesis where components of the review process are simplified to produce a visual and quantitative representation of the scientific evidence from which to commission further review and/or primary research by identifying gaps in research (1). This form of evidence mapping is designed to improve or enhance efficiencies, while still utilizing rigorous, transparent, and explicit methodological approaches, many of which draw from systematic review practices. However, unlike a systematic review, the rEM process does not include steps to assess included studies for quality or risk of bias (i.e., internal validity) and study results are not combined in a meta-analysis. This enables a broader consideration of user needs, such as adhering to more urgent deadlines or allowing accommodations for larger bodies of literature where detailed synthesis is not necessary. The intent of an rEM is to create a robust evidence map of the scientific literature with the assistance of specialized software tools and accomplish it faster than the time typically required to create a traditional evidence map or scoping study.

The scale of the challenge for conducting an rEM depends on the scope of the question(s) being addressed and the associated literature corpus, which may vary considerably by topic. A typical review may require screening thousands or tens of thousands of articles and can utilize hundreds of person-hours of labor. rEMs can generally be completed in a fraction of the time required for a standard systematic review. Depending on the scope of the question being addressed, the time required to complete a typical rEM can be measured in weeks or months compared to systematic reviews, which often take 1–2 years or even longer. Given the challenges of trying to keep up with rapidly-growing bodies of literature, the rEM methodology is a valuable tool that can be applied to a wide range of topics in clinical, environmental health, and related scientific disciplines.

In this study, we sought to generate an rEM in order to comprehensively inform areas that impact COVID-19 outcomes. Our main goals were: (1) to utilize a customized version of our rEM methodology to quickly summarize the evidence of COVID-19 risk and protective factors and susceptible sub-groups; (2) to identify areas where the greatest availability of scientific evidence exists, potentially supporting further investigation such as a systematic review; and (3) to identify areas where evidence is lacking and further research may be warranted. Our results increase the understanding of COVID-19 outcomes that are relevant to public health, while providing an applicable methodology for updating the base of knowledge as the pandemic evolves.

## Methods

**Figure 1:**
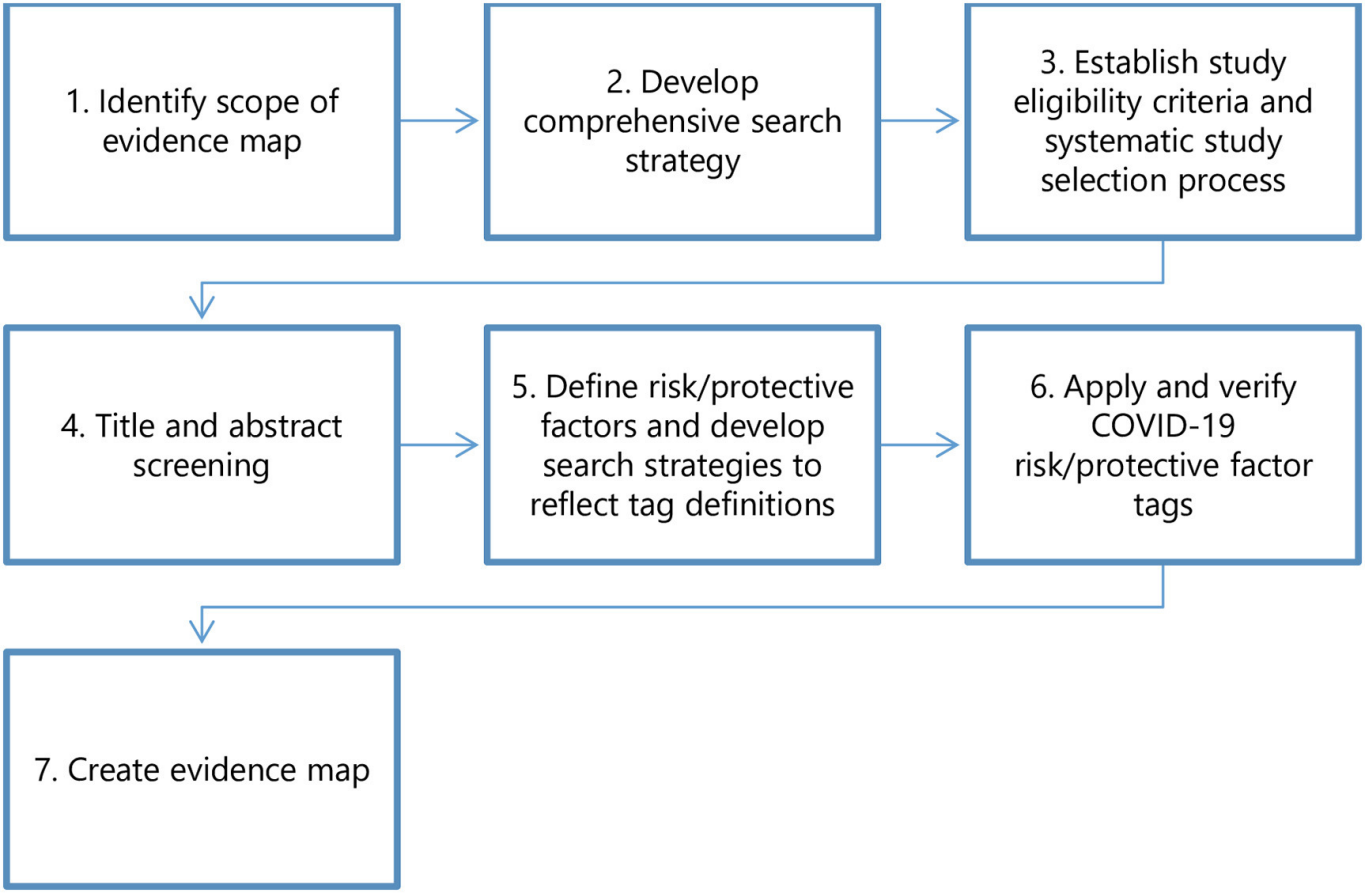
The rEM process. The protocol for this study included a seven-step process based on our previously published methods (1). For Step two, we utilized the existing CORD-19 dataset (https://www.semanticscholar.org/cord19/download) instead of developing a comprehensive search strategy.

We followed a framework similar to our previously published, proof-of-concept application of the rEM methodology to a case study of low-calorie sweeteners and adverse health outcomes (1). An analogous seven-step process (Figure 1) was used to conduct the rEM, with modifications specific to this study question: (1) identify the scope of the evidence map; (2) develop a comprehensive search strategy; (3) establish study eligibility criteria and a systematic study selection process; (4) carry out title and abstract screening; (5) define risk and protective factors and develop search strategies to reflect tag definitions; (6) apply and verify risk/protective factor tags; (7) create the evidence map. We made a minor modification to the process in Step 2: instead of developing a comprehensive search strategy, we utilized an existing dataset, the CORD-19 dataset (https://www.semanticscholar.org/cord19/download). We developed our study protocol and established a PECO (Participants, Exposure, Comparator, and Outcomes) statement that outlined each of these steps before implementing the rEM. Before publishing the protocol on the Open Science Framework, we sought feedback from colleagues in the systematic review field. We incorporated their feedback through multiple iterations before registering the final protocol. In the following sections, we describe each of the seven steps in detail.

### Step 1: Identify the Scope of the Evidence Map

To establish the scope of the evidence map, we developed a study goal, study questions, and PECO statement, which were designed to provide a framework for development of the primary review (7). These elements were outlined beforehand in our publicly available protocol published in the Open Science framework (available at: https://doi.org/10.17605/OSF.IO/XPCKB).

**Study Goal**: To create an evidence map that classifies COVID-19 risk and protective factors, susceptible sub-groups, and the intersections of these categories.

**Study Questions**:

1. What risk factors in human populations are reported in recently published (January–April 2020) COVID-19 literature?
2. What protective factors (i.e., factors reported to lead to better outcomes for those who contract COVID-19) are reported in recently published (January–April 2020) COVID-19 literature?

**PECO statement**:

Participants: Humans
Exposure: COVID-19 virus
Comparator: Compare humans who contracted COVID-19 vs. those who do not or severity of COVID-19 outcomes among those who contracted COVID-19
Outcome: Avoiding contracting the virus or more favorable vs. less favorable outcomes after contracting COVID-19.

### Step 2: Develop Comprehensive Search Strategy

In this step, we would typically develop a detailed strategy to conduct a comprehensive search of the literature. However, given the open access availability of a comprehensive literature set on COVID-19 (Open Research Dataset: CORD-19, search conducted on April 3, 2020), we decided to utilize this existing dataset. This dataset is hosted by Semantic Scholar in collaboration with the Allen Institute for AI and is updated on a weekly basis, with literature included from PubMed, the World Health Organization (WHO) COVID-19 database of publications, the Chan Zuckerberg Initiative, Elsevier open access data, and preprints from medRxiv and bioRxiv (8). By searching these six data sources, the dataset attempts to provide a complete collection of the literature. However, due to the novelty of COVID-19, and a lack of standard terminology at the time, it cannot be ruled out that some relevant references were missed.

### Step 3: Establish Study Eligibility Criteria and a Systematic Study Selection Process

We developed inclusion/exclusion criteria (Table 1) aligning with the overarching study goal, questions, and PECO statement, and outlined these beforehand in the study protocol (Supplementary Data Sheet 1). Studies were included if they were: (1) research involving human subjects; (2) English publications; (3) original sources of new data (including case studies); (4) published between January 1–April 3, 2020; and (5) reporting at least one COVID-19 risk or protective factor. Conversely, studies were excluded if they were: (1) animal or *in vitro* cell studies; (2) non-English studies; (3) non-original sources of data (i.e., reviews, interviews, bibliographies, letters, guidelines, systematic reviews, and meta-analyses); (5) not reporting any COVID-19 risk or protective factors. This decision to exclude studies published prior to 2020 was driven by the fact that the COVID-19 outbreak was first reported in late December 2019 (9).

**Table 1:** Study eligibility criteria.

**Inclusion Criteria**	**Exclusion Criteria**
Human studies	Animal or *in vitro* studies
English language	Non-English language
Original sources of new data (including case studies)	Non-original sources of data (i.e., reviews, interviews, bibliographies, letters, or guidelines; systematic reviews, meta- analyses)
Published between January 1-April 3, 2020	Published prior to January 1, 2020
Report at least one risk or protective factor related to the COVID-19 outbreak	Not reporting any COVID-19 risk or protective factors

*The inclusion and exclusion criteria used for the screening process of titles and abstracts in SWIFT-Active Screener*.

Any factor noted in the literature as decreasing the likelihood of contracting COVID-19 or leading to better COVID-19 outcomes was classified as a protective factor. Any factor noted in the literature as increasing the likelihood of contracting COVID-19 or leading to poorer COVID-19 outcomes was classified as a risk factor. Any risk factor noted can potentially also confer a protective effect [e.g., *gender* was noted in the literature as both a risk factor (for males) and protective factor (for females)].

### Step 4: Carry Out Title and Abstract Screening

Literature screening was conducted using SWIFT-Active Screener (https://www.sciome.com/swift-activescreener/) (10), a web-based, collaborative systematic review software application that automatically prioritizes articles as they are reviewed to increase screening efficiency. During screening, as articles are included or excluded, an underlying statistical model automatically computes which of the remaining unscreened documents are most likely to be relevant. This “Active Learning” model is continuously updated during screening, improving its performance with each article reviewed. Meanwhile, a separate statistical model estimates the number of relevant articles remaining in the unscreened document list. The combination of the two models allows users to screen relevant documents sooner and provides them with accurate feedback about their progress. As a result, the majority of relevant articles can be discovered after reviewing only a fraction of the total number of abstracts, which can result in significant time and cost savings, particularly for large projects. A team of reviewers with topic-related expertise screened references for inclusion. Two reviewers independently screened titles and abstracts in duplicate for the first 100 studies as a pilot test in order to: calibrate screening accuracy between reviewers; discuss conflicts; identify any additional screening questions; and resolve conflicts between reviewers via arbitration by a third systematic review expert. Based on the initial pilot testing, we made the following clarifications to the study eligibility criteria and modified the protocol to reflect these changes:

- Titles and abstracts that mentioned COVID-19 but did not specifically mention risk or protective factors would be excluded;
- Titles and abstracts that mentioned risk factors that occurred after COVID-19 infection and that were not pre-existing risk factors (for example acute renal injury or ground-glass opacity in lung imaging) would be excluded;
- Titles and abstracts that mentioned Severe Acute Respiratory Syndrome (SARS-CoV) or Middle East Respiratory Syndrome (MERS-CoV) viruses but did not mention COVID-19 would be excluded.

Subsequent to completing the pilot testing, the remaining references were single-screened (i.e., each reference was screened by only one reviewer). Titles and abstracts were screened until an estimated 99% recall was achieved—in other words, until the machine learning algorithm predicted that we had identified at least 99% of all relevant (or “included”) references. In our previous work, we have demonstrated that this estimated recall tends to be conservative (10), thus offering high confidence in the stopping criteria. Full texts were not utilized for title and abstract screening, although we did review full text documents for all 217 included articles as an extra validation step to verify that included references were relevant.

### Step 5: Define Risk and Protective Factors and Develop Search Strategies to Reflect Tag Definitions

We developed four broad susceptibility categories based on validated, commonly noted risk factor definitions (11–13) (Table 2). Within these broader categories, we included various subcategories which are also based on validated, commonly noted topics (11–13). Susceptibility categories and subcategories are as follows: (1) *Behavioral Factors* (individual behavioral factors or conscious choices related to lifestyle) with subcategories *Addiction, Nutrition and Diet, Physical Activity, Vaccinations, Sexual Behavior*, and *Medication*; (2) *Physiological Factors* (factors related to body or biology, including genetics) with subcategories *Body Weight, High Blood Pressure, High Cholesterol, High Blood Sugar, Mental Health and Coping, Blood Type, Pregnancy, Hormones, Underlying Health Conditions*, and *Genetic*; (3) *Demographic Factors* (factors related to the overall population, such as age, gender, socioeconomic status) with subcategories *Age, Gender*, and *Socioeconomic*; (4) *Environmental Factors* (broad range of environmental factors, including social, cultural, physical, biological, chemical factors) with subcategories *Infrastructure, Occupation, Living Conditions, Environmental Pollution, Weather*, and *Social Factors*.

**Table 2:** COVID-19 susceptibility categories and number of tagged references^a^^,^^b^.

**COVID-19 Susceptibility Categories and Subcategories**	
Behavioral (*n* = 12) *Individual behavioral risk factors; conscious choices related to lifestyle that could potentially be eliminated*
	Medication (*n* = 5)
	Addiction (*n* = 5)
	Nutrition and diet (*n* = 1)
	Vaccinations (*n* = 1)
	Sexual behavior (*n* = 0)
	Physical activity (*n* = 0)
Physiological (*n* = 107) *Factors directly related to body or biology, including genetics*
	Underlying health conditions (*n* = 81)
	High blood pressure (*n* = 17)
	Body weight (*n* = 2)
	Pregnancy (*n* = 2)
	Genetic (*n* = 2)
	High blood pressure (*n* = 1)
	Blood type (*n* = 1)
	Hormones (*n* = 1)
	High cholesterol (*n* = 0)
	High blood sugar (*n* = 0)
Demographic (*n* = 136) *Factors related to characteristics of the overall population*
	Age (*n* = 98)
	Gender (*n* = 31)
	Socioeconomic (*n* = 7)
Environmental (*n* = 106) *Covers a broad range of topics related to the environment, including social, cultural, political and economic factors, as well as physical, biological, and chemical factors*
	Social factors (*n* = 56)
	Weather (*n* = 22)
	Infrastructure (*n* = 15)
	Occupation (*n* = 10)
	Living conditions (*n* = 3)
	Environmental pollution (*n* = 0)

*The four broad susceptibility categories and subcategories, as well as the number of documents tagged in each category*.

a *Broad susceptibility categories and subcategories were based on validated, commonly noted risk factor definitions (11–13)*.

b *n refers to the number of tagged references in each susceptibility category (i.e., Behavioral, Physiological, Demographic, Environmental, and their subcategories). Each reference can be tagged with one or more categories*.

Given this set of categories, we next designed risk/protective factor search strategies (Supplementary Data Sheet 2) to allow for efficient tagging of references in SWIFT-Review (Sciome Workbench of Interactive computer-Facilitated Text-mining), a freely available, interactive text mining, and machine learning software application (https://www.sciome.com/swift-review/). SWIFT-Review provides tools to assist with searching, categorization, and pattern visualization in literature search results, utilizing statistical modeling, and machine learning methods (14).

First, we created an initial set of keywords for COVID-19 risk and protective factors for each subcategory using keywords developed by examining relevant Medical Subject Heading (MeSH) search terms used to categorize literature at the National Library of Medicine. Although the CORD-19 dataset is not indexed according to MeSH, we were nevertheless able to use the MeSH terminology as a basis for developing corresponding free text search terms. These searches were executed in SWIFT-Review, with iterative refinement as described in Step 6, to label the titles and abstracts of all included references and assign relevant risk/protective factor tags.

### Step 6: Apply and Verify Risk/Protective Factor Tags

We imported the included titles and abstracts into SWIFT-Review to identify COVID-19 risk and protective factors, susceptibility categories and susceptible sub-groups. We used the following iterative procedure for the purpose of “tagging” articles (classifying articles according to a set of predefined categories, in this case COVID-19 risk and protective factors):

a) We used the search feature of SWIFT-Review and the search strategies listed in Supplementary Data Sheet 2 to query the titles and abstracts of all included references. Matching references were provisionally classified according to the corresponding risk and protective factors.
b) The titles and abstracts of all tagged studies were manually reviewed to confirm appropriate tagging. Two screeners read the title and abstract of each reference to confirm that the automatically applied tag was appropriate, remove incorrect tags from studies, and add appropriate tags that were not automatically identified. For example, if an abstract was automatically labeled with the Demographic Category risk factor “age” because it mentioned age of study subjects, but a manual review of the reference identified that it did not mention age in the context of a risk factor associated with COVID-19, the tag in SWIFT-Review was subsequently removed.
c) SWIFT-Review was used to compute term frequency-inverse document frequency (TF-IDF) scores for each set of correctly labeled documents and to identify and rank additional potential keywords for enrichment (i.e., “fingerprint analysis”) in studies labeled with each COVID-19 susceptibility category. TF-IDF scores are a statistical measure used to evaluate how important a term is within a document or literature corpus. Terms having high TF-IDF scores for a particular subset of documents are good candidates for sensitive and precise search terms as they are enriched in that subset relative to the remainder of the dataset.
d) After adding any additional search terms identified in step c above, steps a-c were repeated as necessary until additional search terms no longer provided further benefit with regard to tagging additional studies. This process iteratively created tags (search keyword based tags), which are available both in the final SWIFT-Review Project (available at https://www.sciome.com/rem/) and also described in Supplementary Data Sheet 2.

### Step 7: Create the Evidence Map

After curating, categorizing and summarizing the data as described above, we generated an evidence map describing risk and protective factor characteristics. We used tools in SWIFT-Review to automatically generate frequency tables reporting on the number of studies falling within each risk/protective factor classification.

## Results

**Figure 2:**
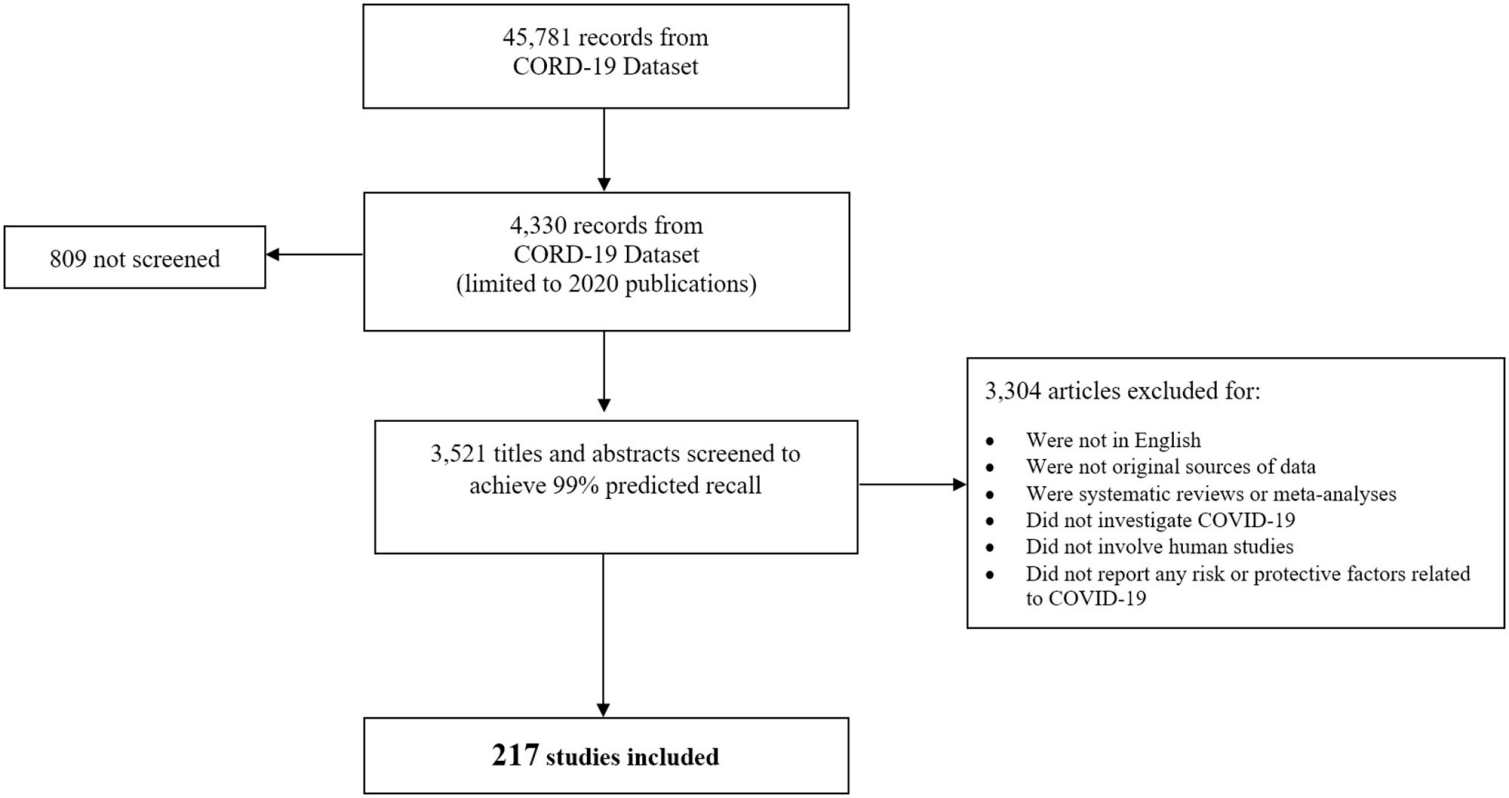
PRISMA diagram screening studies for inclusion. The diagram depicts the flow of reports included in the different phases of screening. Of the 45,781 records available in the CORD-19 dataset as of 4/3/2020, a total of 4,330 records were published in 2020. Among these, 3,521 were screened in SWIFT Active Screener (https://www.sciome.com/swift-activescreener/) (10) to achieve 99% predicted recall. A total of 217 studies met our inclusion criteria and were included in the rEM.

As shown in the PRISMA Diagram (15) (Figure 2), 45,781 records were identified via the CORD-19 dataset. Restricting the dataset to records published January 1—April 3, 2020 reduced the number of studies to 4,330 records. We screened 3,521 (81%) titles and abstracts to reach an estimated 99% predicted recall; 809 articles were not screened. Among the articles screened, 3,304 articles were excluded and 217 relevant studies were included. Because our literature search was conducted in early 2020 during the initial stages of the pandemic, we made the decision beforehand to include pre-prints (articles that have not yet been peer reviewed) in order to fully capture as much relevant scientific information as possible. The aim of our rEM was to provide an initial overview of where research has been conducted and which outcomes were investigated. This information can therefore serve as guidance for further research, possibly including closer examination of the identified studies in the form of targeted systematic review. These results are not intended to, nor should they be considered, as definitive findings that are meant to guide clinical or policy decisions.

**Figure 3:**
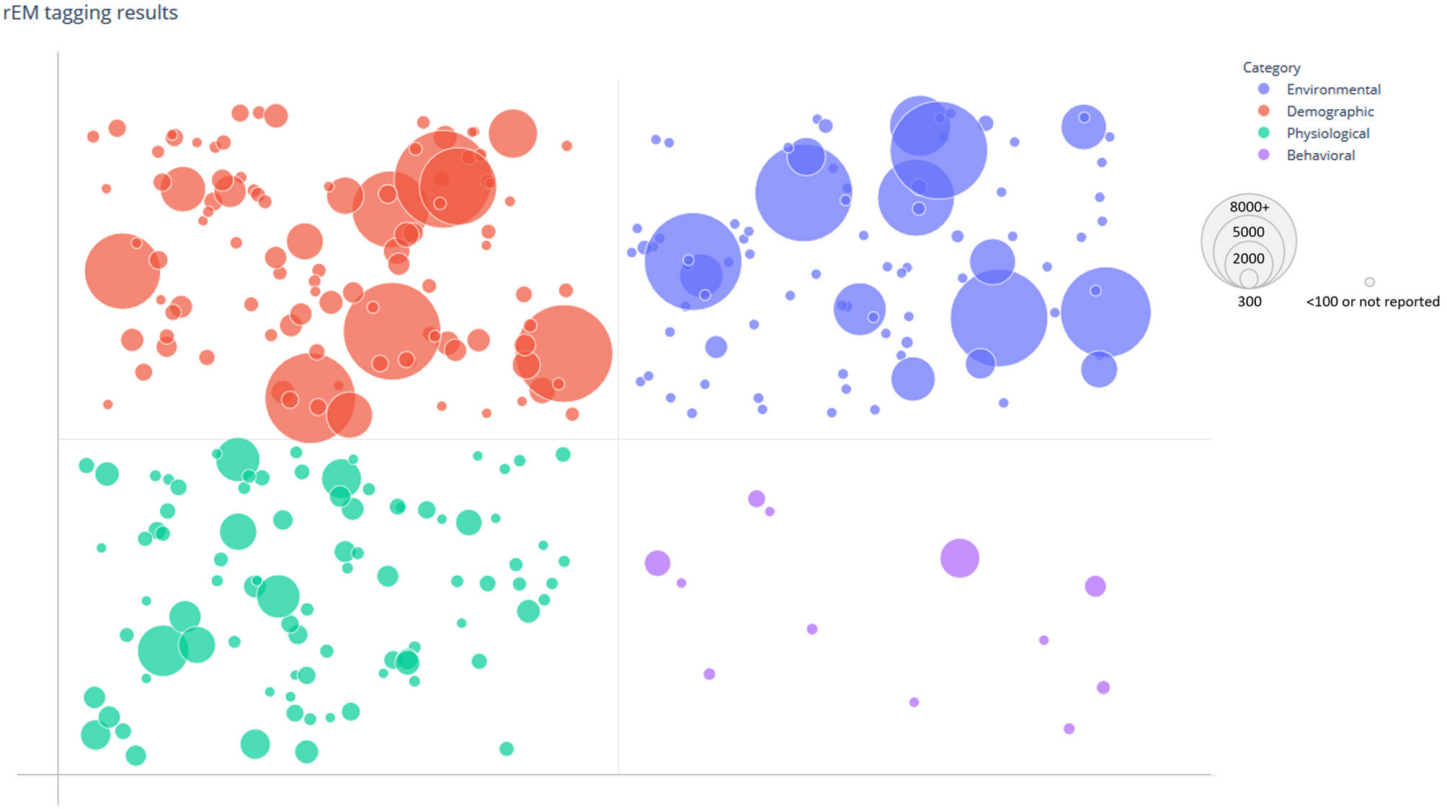
Bubble plot of tagged susceptibility categories. The distribution of categories among the 217 included studies is shown. The data points are grouped and plotted according to tagged risk/protective factors by category (Behavioral, Physiological, Demographic, and Environmental) and study size. Each data point represents a single study and is randomly scattered in each grid to improve visualization of the bubble. The size of each bubble indicates the sample size of the corresponding study with larger bubbles representing larger study sample size.

Included studies were organized and tagged according to each of the established COVID-19 susceptibility categories (Table 2). Tagging was completed using automated searching and categorizing tools available in SWIFT-Review to first automatically tag articles, followed by manual verification of applied tags for quality assurance/quality control (QA/QC). The number of individual studies tagged in each susceptibility category was generally small, particularly in the Behavioral Category (*n* = 12 tagged studies). There were larger numbers of studies tagged in the Physiological (*n* = 107), Demographic (*n* = 136), and Environmental (*n* = 106) categories, specifically those regarding “Underlying Conditions,” “Age,” “Gender,” and “Social Factors.” The distribution of studies among the four broad susceptibility categories can be visualized according to study sample size in a bubble plot (Figure 3). Notably, in addition to having fewer studies, the Behavioral Category generally had smaller sample sizes.

As noted earlier in Section apply and verify risk/protective factor tags (Step 6), the original search terms provided in Supplementary Data Sheet 2 were used to generate tags in SWIFT-Review, and after use of keyword enrichment (fingerprint analyses), “ACE2” was added to the Medication Behavioral susceptibility subcategory.

A common theme among the recently published literature investigating COVID-19 in humans was that older males with multiple comorbidities, particularly hypertension, cardiovascular disease, and diabetes, were shown to be more at risk than the general population. Much of the literature also examined the effectiveness of mitigation strategies (such as social distancing) to slow or prevent the spread of COVID-19.

Despite the body of evidence regarding risk factors, there were few studies examining COVID-19 protective factors. There was also a lack of information regarding the following risk factors: Physical Activity, Sexual Behavior, High Cholesterol, High Blood Sugar, Mental Health and Coping, Race, and Environmental Pollution. Additional details regarding specific risk and protective factor categories are discussed below.

### COVID-19 Risk Factors

COVID-19 risk factors across all categories (i.e., Behavioral, Physiological, Demographic, and Environmental) are summarized below. These include: Age (*n* = 93), Underlying Health Conditions (*n* = 77), Social Factors (*n* = 42), Gender (*n* = 29), High Blood Pressure (*n* = 17), Infrastructure (*n* = 10), Occupation (*n* = 10), and Weather (*n* = 9). Less frequently noted risk factors include: Socioeconomic (*n* = 5), Addiction (*n* = 4), Medication (*n* = 4), Body Weight (*n* = 2), Genetic (*n* = 2), Pregnancy (*n* = 2), Living Conditions (*n* = 2), and Nutrition and Diet (*n* = 1), Vaccinations (*n* = 1), Blood Type (*n* = 1), High Blood Sugar (*n* = 1), and Hormones (*n* = 1). The majority of references were tagged in multiple susceptibility categories (Supplementary Data Sheet 3).

#### Risk Factors in the Behavioral Category

The most commonly noted COVID-19 risk factors in the Behavioral Category were Addiction, Medication, Nutrition and Diet, and Vaccinations (Table 2 and Supplementary Data Sheet 4). There were no relevant references that included Physical Activity or Sexual Behavior risk factors in our review of the literature. The number of studies included in the Behavioral Category was the smallest relative to the three other susceptibility categories for risk factors.

Key findings from the Behavioral Category risk factors include:

- Angiotensin-converting enzyme 2 (ACE2) expression in lower airways was increased in patients with COPD and with current smoking (16).
- History of smoking was reported as a factor leading to the progression of COVID-19 pneumonia (17).
- Smoking was associated with poor COVID-19 outcomes (i.e., heart injury signs) (18, 19).
- There was evidence that an antagonistic relationship between Angiotensin II receptor blocker anti-hypertension drugs and ACE inhibitors may exist, or that individuals with hypertension alone may be less susceptible/had better outcomes than individuals that were hypertensive as well as diabetic and on ACE inhibitors (20, 21).
- Cancer patients receiving treatment were at increased risk of contracting COVID-19 (22, 23).
- Malnutrition was a factor in severe COVID-19 cases in one study (24).
- Bacillus Calmette-Guerin (BCG) childhood vaccination may provide protection to the vaccinated elderly population in countries with BCG vaccination policies (25).

#### Risk Factors in the Physiological Category

A large number of studies investigated characteristics of risk factors in the Physiological Category (*n* = 107), which is the second largest of the four risk categories (Table 2 and Supplementary Data Sheet 4). In this category, the most commonly noted COVID-19 risk factors were Underlying Health Conditions (*n* = 81) and High Blood Pressure (*n* = 17). Less frequently noted risk factors included: Body Weight, Genetic, Pregnancy, Blood Type, High Blood Sugar, and Hormones. We did not identify any relevant references that included High Blood Cholesterol or Mental Health and Coping risk factors.

Key findings from risk factors in the Physiological Category include:

- Individuals with comorbidities such as hypertension, coronary heart disease, diabetes, chronic obstructive pulmonary disorder (COPD), active or latent tuberculosis, cancer, hepatitis B, anorexia were demonstrated to be more likely to contract COVID-19 and to have more severe cases of COVID-19 (16, 19, 26–31).
- Hypertension and diabetes were frequently mentioned in the literature as being common comorbidities associated with negative COVID-19 outcomes.
- Older patients with comorbidities (particularly males) were more susceptible to respiratory failure, to developing severe disease, and were at increased risk of death from COVID-19 (32, 33).
- Patients with multiple comorbidities had poorer COVID-19 outcomes (34, 35).
- Two studies (36, 37) noted that Body Mass Index (BMI) was one of several factors associated with severe COVID-19.
- ACE2 was highly expressed in patients with hypertension, diabetes, and COPD, and patients with such comorbidities may have higher chances of developing severe COVID-19 (16, 38).
- Pregnant women may be more susceptible to COVID-19 (39).
- One study noted that people with blood type A were at higher risk for contracting COVID-19, and that people with blood type O had lower risk of contracting COVID-19 (40).
- One study noted that menopause was an independent risk factor for COVID-19. Estradiol and Anti-Müllerian Hormone were negatively correlated with COVID-19 severity, likely due to their regulation of cytokines related to immunity and inflammation (41).

#### Risk Factors in the Demographic Category

The largest number of studies investigated characteristics of risk factors in the Demographic Category (*n* = 136) (Table 2 and Supplementary Data Sheet 4). In this category, the most commonly noted COVID-19 risk factors were Age (*n* = 98) and Gender (*n* = 31). A smaller number of studies investigated Socioeconomic factors (*n* = 7). In our rEM, there were no studies addressing Race as a demographic factor, likely because of the time frame of the literature (January 1-April 3, 2020), which was prior to extended worldwide spread.

Key findings from risk factors in the Demographic Category include:

- Advanced age and male gender were consistently cited as a risk factor associated with severe COVID-19 outcomes, and older males (>60), particularly those with comorbidities, were most frequently noted as being at greatest risk of contracting COVID-19 and having the poorest outcomes (42– 47).
- The highest mortality risk was noted among the elderly (>80) (42, 48).
- Few studies investigated COVID-19 risk factors in children, and those that did noted that COVID-19 cases in children are typically less severe than those in adults (42, 49).
- Socioeconomic factors were indicated to play a role in mediating local transmission of COVID-19 (50).

#### Risk Factors in the Environmental Category

The Environmental Category (*n* = 106) (Table 2 and Supplementary Data Sheet 4) was the third largest of the four susceptibility categories. The most commonly noted risk factors in this category were Social Factors (*n* = 56), Infrastructure (*n* = 22), Occupation (*n* = 15), and Weather (*n* = 10). A small number of studies investigated Living Conditions (*n* = 3). We did not identify any relevant references that included Environmental Pollution risk factors.

Key findings from risk factors in the Environmental Category include:

- Social Factors were shown to play an important role with regard to COVID-19 spread and containment (51, 52). If social distancing, shutdowns, and other measures (i.e., school closures, isolation, quarantine, use of personal protective equipment, etc.) were implemented early, they reduced the spread of COVID-19, and if they were not properly implemented, they led to increased spread of COVID-19.
- Social distancing and isolation were effective mitigation strategies but also led to family cluster cases if not implemented properly (53, 54).
- Shutdown measures led to a trend toward slower growth of COVID-19 cases; however, this trend was easily reversed if measures were abandoned too early (55, 56).
- In one study (57), healthcare resource availability (in the form of makeshift hospitals) was noted as a protective factor and led to increased COVID-19 survival rates.
- Healthcare workers, medical staff, caretakers, and other occupations with frequent public contact and physical proximity were at greater risk of contracting COVID-19 (34, 58, 59).
- A number of studies suggested that specific weather conditions (ambient temperature, wind speed, humidity) may have an impact on the spread of COVID-19 (60–66).
- Living conditions (i.e., cramped living quarters, limited sanitation) were also shown to play an important role in the spread of COVID-19, particularly among migrants, refugees, cruise ship passengers, etc. (67–69).

### COVID-19 Protective Factors

The majority of studies examined risk factors for COVID-19, and we noted an overall lack of protective factors reported in our review of the literature. A small number reported several factors with protective effects, but not in sufficient numbers to create a visual display of frequency counts. It is important to note that our rEM included pre-prints (articles not yet peer reviewed). We also anticipate that given the rapidly-changing state of the COVID-19 literature base, protective factors reported in the literature may evolve over time and may differ from what was suggested in the earliest publications. Furthermore, because our rEM did not include assessment of the quality of included studies or assessment of risk of bias, the strength and reliability of studies reporting on protective factors is unknown. That is, these studies have not been evaluated for internal or external validity and the factors identified below should not be interpreted as causally associated with protecting against health impacts from COVID-19. Key findings from the COVID-19 Protective Factors include:

- Young age may confer a protective effect against COVID-19, as children and younger people are reported to have less severe outcomes when they contract COVID-19 (42, 49, 70, 71).
- Bacillus Calmette-Guerin (BCG) childhood vaccination was postulated to confer a protective effect against COVID-19, as BCG vaccination was reported in one study to offer protection against other respiratory infections (25).
- People with blood type O were shown in one study to have lower risk of contracting COVID-19 (40).
- Estradiol and Anti-Müllerian Hormone were shown in one study to be protective due to their regulation of cytokines (41).
- Although not demonstrating cause and effect, increased outdoor temperature was associated with lower incidence of COVID-19 (61, 62, 72–74).

### COVID-19 Susceptible Sub-groups

In our rEM, the vast majority of studies indicated that susceptible sub-groups were the elderly, specifically older males with comorbidities. In addition, a small number of studies investigated the increased susceptibility of cancer patients, migrants, refugees, healthcare workers, pregnant women, and children. Reasons for increased risk in these groups may include both medical (weakened immune system) and environmental (living conditions) factors.

## Discussion

The evidence map we have presented provides an overview of risk and protective factors in the early COVID-19 literature and highlights susceptible sub-groups who may have poorer COVID-19 outcomes. To our knowledge, this is the first evidence map that explores the available scientific literature related to risk and protective factors for COVID-19. By utilizing an rEM approach, we were able to provide a comprehensive overview of the recent scientific literature in a time-efficient manner, while still utilizing rigorous, transparent, and explicit methodological approaches. The resulting evidence map is intended to be used as a tool to inspect the available body of evidence, which may inform the design of further detailed reviews (such as a systematic review), as well as guide the focus of new research in areas where knowledge is currently limited. Our results also may directly contribute to public health research by supporting the understanding of factors that impact COVID-19 spread.

We found that recently published literature investigating COVID-19 in humans identifies older males with multiple comorbidities, particularly hypertension, cardiovascular disease, and diabetes as more at risk than the general population. Much of the literature also examines the effectiveness of mitigation strategies (such as social distancing) to slow or prevent the spread of COVID-19. We found a general lack of studies examining COVID-19 protective factors, although we were able to identify some literature suggesting that younger age, Bacillus Calmette-Guerin (BCG) childhood vaccination, blood type O, and Estradiol and Anti- Müllerian Hormone may confer protective effects against contracting COVID-19. In addition, there is some research indicating that increased outdoor temperature may be associated with lower incidence of COVID-19. We identified research areas in which there exists a moderate body of literature where a follow-up review, such as a systematic review, may be informative (i.e., age, gender and comorbidity association with COVID-19) and also areas where evidence is lacking (i.e., risk factors in susceptible sub-groups, risk factors in children, racial disparities in risk, protective factors in general).

We designed and conducted this rEM in 1 calendar month, and an estimated 130 person-hours (screening to 99% estimated recall). While we screened references to 99% estimated recall, we could have saved additional effort if we had stopped at ~95% estimated recall. Only one additional reference was identified after 94.4% estimated recall was achieved; moving from 94.4% estimated recall to 99% recall required screening of 1,238 additional references. Given the novelty and rapid evolution of the available COVID-19 literature, being able to quickly review and summarize these available studies has important implications for policy and decision-making. Because the rEM method makes substantial use of machine learning and information retrieval applications and software, continued development of such tools is likely to further enhance the capacity to perform rEM in an efficient and accurate manner.

We conclude our discussion by noting a few important limitations regarding the resulting evidence map. First, according to our rEM protocol, we dual-screened 100 references (i.e., each reference was reviewed by two screeners) as a pilot to ensure the consistent application of screening criteria. Subsequently, we single-screened the remaining references (i.e., each reference was reviewed by only one screener). Although it is unlikely that a screener errantly included a reference that should have been excluded (given that all references were reviewed during the manual tagging process), it is possible that a relevant reference was erroneously excluded. We also tagged studies based on title and abstract only, and did not extract data from full text documents. While this approach can result in significant time savings, there is a possibility that by tagging studies based on title and abstract alone, we may have, in a few cases, excluded relevant studies or misclassified studies. Furthermore, because we initiated this project in early 2020, during the start of the pandemic, we made the decision beforehand to include pre-prints (articles not yet peer reviewed). Given the novelty of COVID-19 and the time-urgency of the situation, much of the available information at the time had not been peer reviewed, but we still wanted to capture as much of the scientific knowledge that was available on the topic. The intent of this project was not to make judgements or recommendations for clinical practice or policy actions based on the rEM but instead to provide a snapshot of the available evidence to inform future reviews by identifying where research has been conducted and which outcomes have been investigated. As discussed below, we anticipate that the conclusions of an evidence map may change over time when incorporating newer scientific information, particularly when more peer-reviewed literature become available. However, our work illustrates how quickly the evolving landscape of scientific knowledge may be surveyed in a systematic fashion and applied in a useful manner to inform subsequent research activities.

In addition, like most other evidence maps, our rEM does not include quality or risk of bias appraisal of the included studies. Instead, the rEM is designed to identify areas where sufficient evidence exists that can inform scoping and problem formulation for a future systematic review, during which a more formal evaluation of study quality and inferences for policy- and decision-making may be made. In addition, our manual review of tagging results by two independent reviewers did lead to some discrepancies that were discussed and resolved by our team; however, there is some degree of subjectivity in this process, as some tags can be interpreted differently by different reviewers. Finally, as the COVID-19 literature base is evolving very rapidly, reported risk and protective factors may change as new studies are published (e.g., as of May 2020, over 15,000 articles have been added to the CORD-19 dataset since we downloaded references on April 3, 2020). For example, racial disparities in COVID-19 outcomes have been noted in a body of recently published studies (75–77); however, our literature set (focused on literature primarily coming out of China in early 2020) did not cite race as a risk factor. Furthermore, with the increased availability of literature, it is likely that the number of peer-reviewed manuscripts is higher than when we initiated this review. Ideally, this evidence map could be updated on an ongoing basis to incorporate newer scientific evidence as it becomes available, and used to continually monitor what we know about the potential risk and protective factors of COVID-19 as scientific discoveries evolve. This would benefit medical practitioners, policy- and decision-makers in their efforts to stay up-to-date with the latest scientific knowledge, inform where a systematic review of the scientific information might be useful, and guide the development of future research projects, particularly in critical fields where information is currently lacking. To aid with these potential future applications, we have made the latest version of the evidence map, including the SWIFT-Review project file and references list, publicly available at [https://www.sciome.com/rem/].

## Conclusions

We used rEM to summarize the body of evidence related to COVID-19 risk and protective factors reported in recently published literature. We identified research areas where there exists a moderate body of literature and a follow-up review, such as a systematic review, may be informative (i.e., age, gender and comorbidity association with COVID-19) and also areas where evidence is lacking (i.e., risk factors in susceptible sub-groups, risk factors in children, protective factors in general). The automation technologies applied to this rEM in the screening and tagging process can be used to periodically update this evidence base and track scientific knowledge as it evolves, supporting efforts to better protect the public from COVID-19.

## Data Availability Statement

The raw data supporting the conclusions of this article will be made available by the authors, without undue reservation.

## Author Contributions

The study authors were responsible for the study design, collection, analysis, interpretation of data, and writing of the report. RS, BH, JL, RE, and LS conceived and designed the project. BH, AT, JP, MS, SP, TA, and RS designed and implemented the software. RE, LS, JL, BH, AT, and RS analyzed the data. RE, LS, JL, BH, AT, CN, DT, and RS wrote and revised the manuscript. All authors read and approved the final manuscript.

## Conflict of Interest

RE, LS, JL, BH, AT, CN, JP, MS, SP, TA, DT, and RS were employed by Sciome LLC, which funded this work. The authors developed the software used to screen the literature and analyze the data. The authors designed the study, collected the data, decided to publish, and prepared the manuscript as employees of Sciome.

## Abbreviations

rEM: rapid Evidence Map
COVID-19: novel coronavirus 2019
PECO: Participants, Exposure, Comparator, and Outcomes
CORD-19: COVID-19 Open Research Dataset
MeSH: Medical Subject Headings
TF-IDF: term frequency-inverse document frequency.
