# Supplementary material for: Risk and Protective Factors in the COVID-19 Pandemic: A Rapid Evidence Map
Source: Front Public Health. 2020 Nov 24;8:582205. doi: 10.3389/fpubh.2020.582205 (PMC7732416; doi:10.3389/fpubh.2020.582205)
Supplement: Supplementary file 1 [file Data_Sheet_1.docx]

# Supplementary Data Sheet 1: Protocol for COVID-19 Risk and Protective Factor rapid evidence map

**Rapid Evidence Mapping**

**Project Title**
Rapid evidence mapping of risk factors in the COVID-19 pandemic

**Project Goal**

To create an evidence map that classifies COVID-19 risk and protective factors, susceptible sub-groups, and the intersections of these categories.

**Study Question(s)**

**PECO Statement**

**Participants:** Humans

**Exposure:** Contracting COVID-19

**Comparator:** Humans who contracted COVID-19 versus those who do not or severity of COVID-19 outcomes among those who contracted COVID-19

**Outcome:** More favorable versus less favorable outcomes after contracting COVID-19

**Primary Study Question**

1. What risk factors in human populations are reported in recently published (January-April 2020) COVID-19 literature?
   1. We will capture all risk factors reported in the literature (e.g., health, environmental, behavioral, physiological, demographic, genetic, etc.)

**Secondary Study Question**

1. What protective factors (i.e., factors reported to lead to better outcomes for those who contract COVID-19) are reported in recently published (January-April 2020) COVID-19 literature?
   1. Protective factors are any factors that are reported to lead to improved health outcomes in COVID-19 patients (e.g., healthy lifestyle, high level of lymphocytes, etc.)

**Approach**

*Rapid Evidence Mapping* is an expeditious approach based on rigorous methodology. It can be used to quickly summarize an available body of evidence relevant to a research question, identify gaps in the literature to inform future research, and contextualize the design of a systematic review within the broader scientific literature. This approach significantly reduces human effort but still yields results comparable to those from traditional review methods (Lam et. al. *Environ Int*. 2019 Feb;123:451-458).

**Literature Search**

| Potential literature search database/resources to be used | COVID-19 Open Research Dataset |
| --- | --- |
| Search optimization | Search may be optimized to limit the number of hits (options outlined below) |

We will utilize the COVID-19 Open Research Dataset (CORD-19). This dataset consists of scientific literature relevant to the COVID-19 pandemic. It is hosted by Semantic Scholar and updated in collaboration with the Allen Institute for AI on a weekly basis, containing the latest evidence from PubMed (Appendix 1), the WHO Covid-19 database of publications, the Chan Zuckerberg Initiative, Elsevier open access data, and grey literature from the medRxiv and bioRxiv.

**Inclusion and exclusion criteria**

Studies will be *included* if they:

1) Use human subjects;

2) Are original sources of new data (including case studies);

3) Were published in 2020;

4) Investigate at least one risk factor related to the COVID-19 outbreak;

5) Have English title and abstract available.

Studies will be *excluded* if they:

1. Do not include human subjects (i.e., animal or *in vitro* evidence);
2. Are non-original sources of data, including reviews, interviews, bibliographies, letters, or guidelines;
3. Were published prior to 2020;
4. Do not report any COVID-19 risk factors;
5. Do not have English title and abstract available.

- Titles and abstracts that mention COVID-19 but do not specifically mention risk or protective factors will be excluded; and
- Titles and abstracts that mention risk factors that occurred after COVID-19 infection (were not pre-existing risk factors, for example acute renal injury or ground-glass opacity in lung imaging) will be excluded; and
- Titles and abstracts that mention Severe Acute Respiratory Syndrome (SARS-CoV) or Middle East Respiratory Syndrome (MERS-CoV) viruses but do not mention COVID-19 will be excluded.

**Literature Screening**

| Screening Level | Level 1 screen: Titles and abstracts used for screening |
| --- | --- |
| Screening Approach | Using *SWIFT* *Active Screener* software, we will screen the first 100 references in duplicate for calibration, then single-screen for remainder of references |
| Recall Target | Machine-learning prioritization of references and screening will be conducted up to meeting 99% estimated recall |

Next, we will screen the titles and abstracts of retrieved studies for inclusion and relevance to each study question. We will use SWIFT-Active Screener, a web-based collaborative system, to deduplicate and screen studies for relevance using pre-defined inclusion/exclusion criteria. SWIFT-Active Screener utilizes machine learning to statistically rank articles by relevance, to reduce screening time and effort required to identify the most relevant studies. The final product will be a comprehensive list of included articles relevant to the study questions, based solely on title and abstract screening.

**Analytics and Reporting**We will then search, categorize, analyze, and visualize patterns in the collection of titles and abstracts of included studies. To do this, we will utilize all included titles and abstracts, group and analyze them using SWIFT-Review and its built-in statistical modeling and machine learning tools. In particular, we will apply the tools in SWIFT-Review to automatically identify, recognize, and extract risk and protective factors from the titles and abstracts of included references and summarize and visualize data from included studies, minimizing the need to manually extract data.

Using the aforementioned analysis, we will create a rapid Evidence Map (rEM) that will categorize and visually depict the relevant literature related to risk factors in humans, utilizing semi-automated machine learning approaches to automatically sort, categorize, and tag references.

Outputs from the rEM process will include:

1. Frequency and proportion counts of categorizations within the study questions;
2. Heat/bubble maps visually displaying categorizations within the study questions;
3. Identification of areas with the most scientific evidence, informing where a more thorough review (including full-text review) could be potentially completed;
4. Identification of areas where scientific evidence is lacking and where more research is needed.

**Deliverable**

A comprehensive report (a rapid evidence map) which shall include the aforementioned outputs and analysis.

**Timeline**

The timeline for completion greatly depends on the size of literature set for evidence mapping. A more accurate timelines can be estimated subsequent to finalizing the search strategies.

**References**

COVID-19 Open Research Dataset (CORD-19). 2020. Version 2020-03-20. Retrieved from https://pages.semanticscholar.org/coronavirus-research. Accessed 2020-04-03. doi:10.5281/zenodo.3715505

**Appendix 1: Search**

- 1. **PubMed search strategy used in creation of the CORD-19 corpus**

"COVID-19" OR Coronavirus OR "Corona virus" OR "2019-nCoV" OR "SARS-CoV" OR "MERS-CoV" OR “Severe Acute Respiratory Syndrome” OR “Middle East Respiratory Syndrome”
