# Supplementary material for: Risk and Protective Factors in the COVID-19 Pandemic: A Rapid Evidence Map
Source: Front Public Health. 2020 Nov 24;8:582205. doi: 10.3389/fpubh.2020.582205 (PMC7732416; doi:10.3389/fpubh.2020.582205)
Supplement: Supplementary file 2 [file Data_Sheet_2.docx]

# Supplementary Data Sheet 2: Final Search Terms for COVID-19 Susceptibility Category Tagging

| **COVID-19 Susceptibility Category Final Search Terms** | |
| --- | --- |
| **Behavioral Susceptibility Subcategories** | **Search Terms** |
| **Addiction** | drug abuse, drug dependen*, addict*, substance use disorder*, drug use disorder*, substance dependen*, drug habit*, alcohol*, binge drink*, liquor*, beer*, wine*, vodka, gin, baijiu, tequila, whiskey, brandy, rum, soju, drunkenness, nonsmoke*, smoke* smoking, tobacco, cigarette*, cigar*, nicotine, e-cig*, ecig*, vape, vaping, nicotrol, electronic nicotine, nicotine inhalator*, apnea, respiration, cough*, dyspnea, epistaxis, hemoptysis, hoarseness, hypercapnia, hyperoxia, hyperventilation, hypocapnia, hypoventilation, hypoxia, mouth breathing, respiratory sound*, snoring, sneezing, tachypnea |
| **Nutrition and Diet** | diet*, nutrition*, nutrient*, nutritive*, nourishment*, appetite*, digestion*, hunger, gastrointestinal absorption, gastric absorption, gut absorption, vegetarian*, vegan*, gluten-free, low-fat*, low-carb*, caloric, calories, cook, cooking, eat, fast food, processed food, food intake, ingestion |
| **Physical Activity** | Exercise, fitness, physical activity, physical exertion, physical fitness, physical endurance, physical conditioning, workout*, running, jogging, swimming, walking, aerobic*, sports, baseball, basketball, bicycling, boxing, football, golf, gymnastics, hockey, martial arts, Tai Ji, mountaineering, tennis, skating, skiing, soccer, track and field, volleyball, weight lifting, wrestling |

| **COVID-19 Susceptibility Category Final Search Terms** | |
| --- | --- |
| **Vaccinations** | immunization*, immunological sensit*, immunologic stimulat*, immunostimula*, vaccin*, acquired immunity, protective inoculation* |
| **Sexual Behavior** | unsafe sex, high risk sex, unprotected sex, safe sex, safer sex, protected sex, responsible sex, sexual behavior, sexual activit*, contraception, contraceptive*, inhibition of fertilization, fertilization inhibition, fertility control, birth control, condom*, intrauterine device*, IUD |
| **Medication** | pharmaceutic*, prescrib*, prescription*, nonprescription*, medication, Rx, controlled substance*, dosage*, pharmacotherap*, drug*, therap*, treatment*, **ACE2^+^** |
| **Physiological Susceptibility Subcategories** | **Search Terms** |
| **Body Weight** | body weight, weight gain, weight loss, overweight, corpulent, obese, obesity, underweight, thinness, leanness, adipose, abdominal fat*, intraabdominal fat*, retroperitoneal*, visceral fat*, malnutrition, malnourish*, nutritional deficien*, undernutrition*, body mass index, BMI, Quetelet Index, body fat |

| **COVID-19 Susceptibility Category Final Search Terms** | |
| --- | --- |
| **High Blood Pressure** | hypertension, hypertensive, high blood pressure*, blood pressure*, diastolic pressure*, pulse pressure*, systolic pressure*, arterial pressure*, arterial tension*, aortic pressure*, aortic tension*, sphygmomanometer* |
| **High Blood Cholesterol** | hypercholesterolemia*, high cholesterol*, elevated cholesterol*, high blood cholesterol*, hyperlipidemia*, hyperlipemia*, lipidemia*, lipemia*, cholesterol*, epicholesterol*, lipid disorder* |
| **High Blood Sugar** | hyperglycemia*, glucose intolerance, blood glucose, blood sugar*, glycemic index*, glycemic indice*, hypoglycemia, glucose |
| **Mental Health and Coping** | mental health, mental hygiene, psychological adapt*, psychologic adapt*, coping, adaptive behav*, emotional adjust*, stress*, distress*, suffering*, burnout, fatigue*, post-traumatic, posttraumatic, social adjust*, anxiety, anxieties, nervousness, hypervigilan*, neurotic, panic, phobia*, phobic, depression, depressive, melancholia*, adjustment disorder*, psychological trauma*, resilience*, hopelessness*, hope*, desolation, desperation, loneliness, isolation, alienation, social breakdown, morale*, courage*, bravery, heroism |
| **Blood Type** | blood type*, blood group*, ABO Genotype*, O negative, O positive, A negative, A positive, B negative, B positive, AB negative, AB positive |

| **COVID-19 Susceptibility Category Final Search Terms** | |
| --- | --- |
| **Pregnancy** | pregnan*, gestation, carry a child, obstetric labor*, premature birth*, preterm birth*, premature deliver*, preterm deliver*, premature rupture of fetal membrane*, premature rupture of membrane*, preterm PROM, fetal tachycardia, fetal distress, fetal status, parturient, Apgar score*, neonatal intensive care, NICU, newborn intensive care |
| **Hormones** | gonadal steroid hormone*, sex steroid hormone*, sex hormone*, menstrual cycle*, endometrial cycle*, ovarian cycle*, menopaus*, perimenopause*, postmenopaus*, premenopaus*, hot flash*, estradiol*, E2, oestradiol*, Anti Mullerian Hormone*, Anti Mullerian Factor*, Mullerian Inhibiting Factor*, Mullerian Inhibiting Substance*, Mullerian Inhibiting Hormone*, AMH, Follicle Stimulating Hormone*, FSH, Follitropin |
| **Underlying Health Conditions** | diabetes*, diabetic*, prediabetic, glucose intoleran*, glucose toleran*, OGTT, Cardiovascular*, cardiac*, cardio*, CVD, heart defect*, vascular malformation*, heart disease*, heart failure*, heart valve disease*, myocardial, myocarditis, carditis, pericardial, pericarditis, endocarditis, Heart arrest, heart defect*, pulmonary heart disease*, ventricular dysfunction*, vascular disease*, vascular disorder*, aneurysm*, cerebrovascular*, ischemic, ischemia, embolism, thrombosis, hypertension*, prehypertension*, hypotension, pulmonary, vasculitis, Coronary Disease*, chronic disease*, chronic illness*, chronically ill, chronic condition*, brain disease*, carotid artery disease*, cerebral disease*, cerebral infarct*, subcortical infarct*, choroidal artery infarct*, intracranial arterial disease*, intracranial hemorrhage*, cerebral hemorrhage*, stroke*, brain infarction*, comorbid*, multimorbid*, coexisting condition*, cancer*, neoplasia*, neoplasm*, tumor*, malignan*, precancer*, carcinoma*, adenocarcinoma*, respiratory tract disease*, bronchial disease*, asthma*, asthmatic*, respiration disorder*, underlying disease*, anhelation, shortness of breath, breathing difficult*, renal insufficienc*, kidney insufficienc*, kidney disease*, kidney injur*, kidney failure*, renal disease* AKI, disease*, syndrome*, chronic obstructive pulmonary disease*, chronic obstructive pulmonary disorder*, COPD, chronic obstructive airway disease*, chronic obstructive airway disorder*, COAD, chronic airflow obstruction*, obstructive lung disease*, hepatitis, HepB, HepC, coinfection*, co- infection*, mixed infection*, polymicrobial infection*, secondary infection*, anemia*, hypoproteinemia*, |

| **COVID-19 Susceptibility Category Final Search Terms** | |
| --- | --- |
|  | tuberculos*, TB, neurologic manifestation*, neurologic signs, neurologic symptom*, neurological manifestation*, neurologic deficit*, neurological deficit*, neurologic deficit*, neurologic dysfunction* |
| **Genetic** | genetic research, genetics, genetic disease*, genetic disorder*, genetic mutation*, gene defect*, genetic risk*, genetic factor*, hereditary disease*, cystic fibrosis, mucoviscidosis, Fibrocystic Disease of Pancreas, Pancreas Fibrocystic Disease, sickle cell anemia*, sickle cell disease*, Hemoglobin S Disease*, sickle cell disorder*, sickling disorder*, HbS disease*, immune phenotype, immune profiling, immunological phenotype, lymphopeni*, lymphocytopenia*, lymphocyte*, lymphoid cell*, lymphocyte count*, lymphocyte number*, total lymphocyte* |
| **Demographic Susceptibility Subcategories** | **Search Terms** |
| **Age** | age, ages, aged, ageing, adolesc*, teen*, youth*, minor*, juvenile*, adult*, newborn*”, neonate*, infant*, child*, boy*, girl*, toddler*, under five*, elder*, geriatr*, geront*, senior*, latelife, laterlife, years, young, younger, old, older, underage* |
| **Gender** | gender*, sex role*, male*, men, female*, women*, woman*, femin*, masculine*, transgender*, transsex*, transwoman*, transwomen*, trans woman*, trans women*, transman*, transmen*, trans man*, trans men*, two-spirit person*, sex characteristic*, sex dimorphism, sex difference*, sexual dimorphism, cisgender*, Intersex Person*, homosexual*, bisexual*, queer*, gay*, lesbian*, LGB*, GLBT*, MSM |
| **Socioeconomic** | socioeconomic*, SES, standard of living, living standard*, income, inequalit*, economic status, poverty, social class, salary, salaries, wages, ethnic group*, ethnicity, nationality, |

| **COVID-19 Susceptibility Category Final Search Terms** | |
| --- | --- |
|  | education, educational status, literacy, gross domestic product*, GDP |
| **Environmental Susceptibility Subcategories** | **Search Terms** |
| **Infrastructure** | healthcare, health care, health service*, health plan, patient care, health resource*, hospital resource*, medical resource, resource allocate*, ration*, infrastructure, burden, barrier*, disparity, disparities, equality, inequality, equity, inequities, stockpile*, time to treatment, treatment delay*, delayed treatment, delayed hospitalization, delayed hospitalization, medically underserved, underserved population*, physician shortage* |
| **Occupation** | occupation*, personnel, workforce, manpower, essential worker*, essential employee*, essential staff*, emergency responder*, EMT, first responder*, police*, firefighter*, allied health professional*, healthcare assistant*, healthcare worker*, health care support worker*, paramedic*, health worker*, anesthetist*, anesthesiologist*, physician*, doctor*, coroner*, medical examiner*, medical staff*, hospital staff*, hospitalists, nurse*, nursing, practitioner*, respiratory therapist*, migrant*, farmer*, agricultural worker*, farm worker*, presenteeism, caregiver*, care giver* |
| **Living Conditions** | social condition*, living condition*, poverty, slum, slums, ghetto*, refugee*, favela*, suburban*, suburb*, nonmetropolitan, rural, prison*, penal institution*, detention camp*, detention center*, border camp*, detainment camp*, detainment center*, detention facilit* |
| **Environmental Pollution** | pollution, pollutant*, air quality, environmental exposure*, inhalation exposur*, maximum allowable concentration*, threshold limit value*, hazardous substance*, hazardous material*, toxic environmental*, environmental toxin*, |

| **COVID-19 Susceptibility Category Final Search Terms** | |
| --- | --- |
|  | biohazard*, airborne particulate matter*, ambient particulate matter*, dust, housedust, mold, mould, fungi, fungus |
| **Weather** | weather, humidit*, season*, climate*, climatic*, climatology, biometeorology, winter, spring, summer, temperature*, solar energ*, solar power*, solar radiation*, cosmic radiation*, precipitation, wind, winds |
| **Social Factors** | social factor*, sociological factor*, social characteristic*, social trait*, social attribute*, sociological characteristic*, family relationship*, family separation, intergenerational*, grandparent*, parenting, social conditions*, social environment*, social ecolog*, lifestyle*, life chang*, community network*, social support*, social network*, holiday, holidays, festival*, vacation*, new year*, celebration, social distanc*, public distanc*, close contact*, family outbreak*, news media, communications media, social media, mass media, cultural norm*, transportation, transit, traffic restrict*, traffic control*, airport*, airplane*, trains, ships, cruises, tourist*, travel restrict*, travel ban*, migration restrict*, infection control*, isolation, isolated, isolating, quarantin*, cross infect*, mask, masks, personal protective equipment, PPE, protective clothing*, respiratory protective devic*, N95, respirator use, protective factor*, protective device*, disease eradicate*, disease control*, contact tracing*, contract trace*, disease notification*, exposure notification*, hotline*, call center*, school closur*, closed school*, homeschool*, bans on large gathering*, shutdown*, lockdown*, cluster*, clustering*, exposure to covid-19, covid-19 expos*, person to person, contact mode*, household contact*, contact pattern*, mitigation effort*, incident report*, risk report*, risk management*, risk assessment* |

*The asterisks shown are wildcards used to maximize search results in library databases. Wildcards are used in search terms to represent one or more characters; for example, searching for educat* will find all possible endings of the root word, including educate, educated, education, educational, educator.

^+^Denotes search term added after fingerprint analysis.
