# Supplementary material for: Risk and Protective Factors in the COVID-19 Pandemic: A Rapid Evidence Map
Source: Front Public Health. 2020 Nov 24;8:582205. doi: 10.3389/fpubh.2020.582205 (PMC7732416; doi:10.3389/fpubh.2020.582205)
Supplement: Supplementary file 4 [file Data_Sheet_4.docx]

# Supplementary Data Sheet 4: COVID-19 Susceptibility Subcategory Supplemental Figures

**Figure 1. COVID-19 Behavioral susceptibility subcategories.** The 12 studies addressing behavioral susceptibility subcategories are shown. The number of studies in each subcategory is indicated. A green to white gradient indicates decreasing frequency counts.

| **Behavioral Susceptibility Subcategories** | **Number of Studies** |
| --- | --- |
| Addiction | 5 |
| Medication | 5 |
| Nutrition and Diet | 1 |
| Vaccinations | 1 |
| Physical Activity | 0 |
| Sexual Behavior | 0 |

**Figure 2. COVID-19 Physiological susceptibility subcategories.** The 107 studies addressing physiological susceptibility subcategories are shown. The number of studies in each subcategory is indicated. A green to white gradient represents decreasing frequency counts.

| **Physiological Susceptibility Subcategories** | **Number of Studies** |
| --- | --- |
| Underlying Health Conditions | 81 |
| High Blood Pressure | 17 |
| Body Weight | 2 |
| Genetic | 2 |
| Pregnancy | 2 |
| Blood Type | 1 |
| High Blood Sugar | 1 |
| Hormones | 1 |
| High Blood Cholesterol | 0 |
| Mental Health and Coping | 0 |

**Figure 3. COVID-19 Demographic susceptibility subcategories.** The 136 studies addressing physiological susceptibility subcategories are shown. The number of studies in each subcategory is indicated. A green to white gradient represents decreasing frequency counts.

| **Demographic Susceptibility Subcategories** | **Number of Studies** |
| --- | --- |
| Age | 98 |
| Gender | 31 |
| Socioeconomic | 7 |
| Race | 0 |

**Figure 4. COVID-19 Environmental susceptibility subcategories.** The 106 studies addressing physiological susceptibility subcategories are shown. The number of studies in each subcategory is indicated. A green to white gradient represents decreasing frequency counts.

| **Environmental Susceptibility Subcategories** | **Number of Studies** |
| --- | --- |
| Social Factors | 56 |
| Infrastructure | 22 |
| Occupation | 15 |
| Weather | 10 |
| Living Conditions | 3 |
| Environmental Pollution | 0 |
